# Progesterone attenuates cerebral edema in neonatal rats with hypoxic-ischemic brain damage by inhibiting the expression of matrix metalloproteinase-9 and aquaporin-4

**DOI:** 10.3892/etm.2013.1116

**Published:** 2013-05-15

**Authors:** XIAOYIN WANG, JUNHE ZHANG, YUXIN YANG, WEIHUA DONG, FANG WANG, LI WANG, XIAOJUAN LI

**Affiliations:** 1 Department of Biochemistry and Molecular Biology, Xinxiang Medical University;; 2 Department of Ophthalmology, The First Affiliated Hospital of Xinxiang Medical University;; 3 Department of Physiology and Neurobiology, Xinxiang Medical University, Xinxiang, Henan 453003, P.R. China

**Keywords:** progesterone, hypoxic-ischemic, matrix metalloproteinase, aquaporin, blood-brain barrier, cerebral edema

## Abstract

The aim of this study was to investigate the effects of progesterone (PROG) on blood-brain barrier (BBB) permeability, cerebral edema and the expression of matrix metalloproteinase-9 (MMP-9) and aquaporin-4 (AQP-4) in neonatal rats with hypoxic-ischemic brain damage (HIBD) and to explore the mechanism of its neuroprotective effect. Sixty 7-day-old Wistar rats were divided into sham surgery, hypoxic ischemia (HI) and drug prophylaxis (PROG) groups. HIBD animal models were established. All the animals were sacrificed after 24 h. The BBB was assessed using Evans blue. Cerebral moisture capacity was determined using the dry-wet method. MMP-9 was detected in the brain tissues using enzyme-linked immunosorbent assay. The expression of AQP-4 and MMP-9 in the cerebral cortex was observed using immunohistochemistry and real-time polymerase chain reaction. The MMP-9 levels in the cortex, BBB permeability, cerebral moisture capacity and expression of AQP-4 and MMP-9 in the HI group were significantly higher compared with those in the sham surgery group (P<0.01), and they were significantly lower in the drug prophylaxis group compared with those in the HI group (P<0.05). In conclusion, PROG reduces BBB damage and cerebral edema and inhibits MMP-9 generation to protect rat brains against HIBD. The protective effect of PROG may be correlated with downregulated expression of AQP-4 and MMP-9 in the cerebral cortex.

## Introduction

Hypoxic-ischemic brain damage (HIBD) is a common life-threatening disease during the neonatal period. HIBD caused by asphyxia remains a common cause of numerous types of chronic disability, including cerebral palsy, mental retardation and epilepsy (1,2). HIBD has an incidence of 1–80/1,000, among which 10–20% of patients succumb during the neonatal period (3,4). Among the survivors, 25–30% suffer long-term neurodevelopmental sequelae, which cause huge domestic and social burdens, as well as great pressure on pediatricians (5,6). Further studies on the mechanism of and treatment measures for HIBD are urgently required.

Cerebral edema is the most basic change in the pathophysiology of HIBD. This condition is the primary cause for the development and aggravation of HIBD or even HIBD-induced mortality (7). Therefore, controlling cerebral edema early is critical for HIBD prognosis. At present, a number of theories have been proposed to explain the development of cerebral edema. Matrix metalloproteinases (MMPs) are the most important type of proteinase responsible for extracellular matrix decomposition. MMPs degrade extracellular matrices and destroy the blood-brain barrier (BBB) to participate in the formation of cerebral edema and the occurrence of brain damage. Aquaporins (AQPs) are specific membrane proteins that have been recently studied. AQP-4, an AQP extensively distributed in brain tissues, is considered the most significant (8,9). AQP-4 contributes to cerebral edema following brain ischemia and it is correlated with the development of cerebral edema in brain damage caused by various factors (10). However, the dynamic changes in AQP-4 levels in the brain tissues of HIBD neonatal rats and its role in the development of cerebral edema in brain damage have rarely been reported. Early inhibition of AQP-4 expression in brain tissues may provide a new treatment protocol for cerebral edema.

Progesterone (PROG) is a natural progestin. PROG is generated by endocrine tissues as well as the nervous system and acts on reproductive organs to regulate reproductive function (11,12). However, previous studies have shown that the effects of PROG are not limited to reproduction. It is synthesized and secreted in the nervous system and it affects the structure and functions of the system (13,14). The brain damage-preventive effect of PROG and the associated mechanism of action have attracted increasing attention. PROG protects brain tissue during the development of HIBD; it antagonizes the generation of free radicals, inhibits cell apoptosis and alleviates cerebral edema (11,13). However, the actual brain-protective mechanism remains unclear. Studies concerning the brain-protective effect of PROG have primarily focused on brain damage in adult rats, whereas those on neonatal rats are rarely reported. Whether PROG reduces BBB damage and cerebral edema by affecting the expression of AQP-4 and MMP-9 remains unknown.

## Materials and methods

### Animals and grouping

Sixty 7-day-old Wistar rats weighing 11–19 g were supplied by the Laboratory Animal Centre of Xinxiang Medical University (Xinxiang, China). The rats were randomized into a sham surgery group, hypoxic ischemia group (HI group) and drug prophylaxis group (PROG group). The sham surgery group was merely subjected to a cervical incision (no HI performed). The HI group was managed according to the HI animal model establishment method below. The drug prophylaxis group was intraperitoneally injected with 0.5 g/l PROG solution at a dose of 8 mg/kg, 30 min before anoxia management. This study was performed in strict accordance with the recommendations in the Guide for the Care and Use of Laboratory Animals of the National Institutes of Health. The animal use protocol has been reviewed and approved by the Institutional Animal Care and Use Committee (IACUC) of Xinxiang Medical University.

### Model establishment

Following anesthetization of the neonatal rats with absolute ether, the rats were fixed in the supine position on a rat bench. A midline incision was performed on the cervical skin. The left common carotid artery was separated at the medial aspect of the sternocleidomastoid muscle and then ligated. Following incision suturing, the animals recovered at room temperature for 2–3 h. Then, the animals were placed into a hermetic container at 37°C for HI animal model establishment by introducing a mixture of 8% O_2_ and 92% N_2_ at a rate of 1.5 l/min for 2.5 h (15,16). The sham surgery group was only subjected to the separation of the left common carotid artery without ligation and hypoxia.

### Determination of BBB permeability

BBB permeability was determined by detecting the intracephalic amount of Evans blue (EB) dye. Each animal received 20 g/l EB and physiological saline solution injected into the heart cavity. After 1 h, the eyes and skin turned blue. After another hour, the animals were decapitated. Right-brain tissues were collected, weighed and incubated for 72 h with twice their volume of formamide in a water bath at 50°C. The supernatant was collected and its absorbance was recorded using a spectrophotometer to establish a standard curve. The amount of EB in the test sample was calculated using the standard curve and the results are presented in *μ*g/g wet brain tissue.

### Determining the water content of the brain tissues

The rats were decapitated 24 h after HI and their brain tissues were obtained immediately. The tissues were placed on a culture dish containing a piece of qualitative filter paper soaked in physiological saline to prevent drying. Clean, covered glass weighing bottles were prepared prior to the experiment. Left cortical tissues (∼ 80 mg) were cut and placed into the preweighed bottle to determine the wet weight on an electronic balance. Then, the tissues were dried in an oven at a constant temperature of 110°C for 48 h until a constant weight was reached. The percentage of water content (WC) was calculated based on the Elliot formula: WC (%) = [(wet weight - dry weight)/wet weight] ×100.

### MMP-9 determination

The animals were decapitated 24 h after hypoxia. The left-brain tissues were isolated from the skull and washed with ice-cold physiological saline to remove surface blood. The cortex and hippocampus were separated, blotted, weighed and poured into a homogenizer. The tissues were homogenized with 1 ml ice-cold physiological saline per 100 mg brain tissue. High-speed hypothermal centrifugation was performed for 15 min and the supernatant was obtained. An enzyme-linked immunosorbent assay (ELISA) was performed to determine the MMP-9 content using an ELISA kit (Saimo Biotechnology Co., Ltd., Shanghai, China) on a microplate reader (American Hyperion MR III type; Biotek Instruments Inc., Winooski, VT, USA).

### Immunohistochemistry

The animals were sacrificed 24 h after HIBD and brain tissues were obtained immediately. The tissues were segmented from the optic chiasm and then fixed overnight in 10% formalin. The tissues were routinely dehydrated, cleared, embedded in paraffin, sectioned (5 *μ*m), dewaxed, dried and then stored at 4°C. The tissues were stained immunohistochemically for MMP-9 and AQP-4 using a test kit according to the manufacturer’s instructions (ZSGB-BIO, Beijing, China). The primary antibodies were replaced with phosphate buffer in the negative control. Cells positive for MMP-9 and AQP-4 were those with buffy-stained cytoplasm and membranes. Three brain tissue sections were obtained from each animal. Two random cortical visual fields were observed under a light microscope (magnification, ×400). Digital images were obtained and analyzed for staining using an HMIAS-2000 color image analysis system (Tongji Medical College Clear Screen Imaging Inc., Wuhan, China). The mean optical density was calculated.

### Real-time reverse transcription-polymerase chain reaction (RT-PCR)

Total RNA was extracted from the rat cortex. The sample was reverse transcribed into cDNA using a reverse transcription kit (Takara Bio Inc., Dalian, China). β-actin served as the internal reference. The respective sequences of the upstream and downstream primers of β-actin were: 5′-CGTTGACATCCGTAAAGACC-3′ and 5′-GCTAGGAGC CAGGGCAGTA-3′, with a 260 bp product. The primers for MMP-9 were 5′-TGCGTATTTCCATTCATC-3′ and 5′-CCT TGGGTCAGGTTTAGAG-3′, with a 495 bp product, and those for AQP-4 were 5′-CATCGGAGCTGGGATCCTCTA-3′ and 5′-GAGCTCCACGTCAGGACAGAA-3′, with a 394 bp product. After 2% agarose gel electrophoresis, the grey scales of the PCR amplification products were analyzed using Band Leader 3.0 gel image manipulation software (http://www.bio-soft.net/draw/BandLeader.htm. Accessed December 20, 2012). The relative mRNA levels of the genes of interest are presented as ratios between the band grey scale of their product and that of β-actin.

### Statistical analysis

All data are presented as mean ± standard deviation. Statistical analyses were performed using SPSS 12.0 software (SPSS, Inc., Chicago, IL, USA). A t-test was performed to compare the means of paired samples and one-factor analysis of variance (ANOVA) was used to compare means. P<0.05 was considered to indicate a statistically significant difference.

## Results

### Effect of PROG on MMP-9 in the cerebral cortex

**Figure 1.**
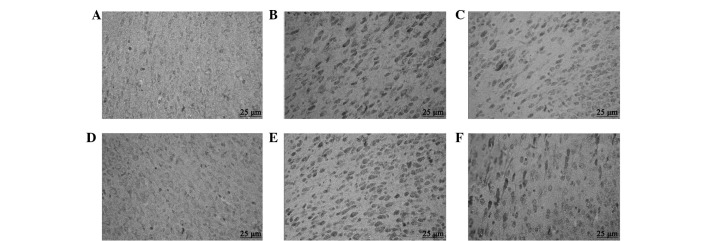
Expression of matrix metalloproteinase (MMP)-9 and aquaporin (AQP)-4 was observed using immunohistochemistry [magnification, ×400; stained with SP (streptavidin-perosidase)]. MMP-9 expression in the (A) sham surgery, (B) hypoxic ischemia (HI) and (C) progesterone groups. AQP-4 expression in the (D) sham surgery, (E) HI and (F) progesterone groups, respectively.

**Figure 2.**
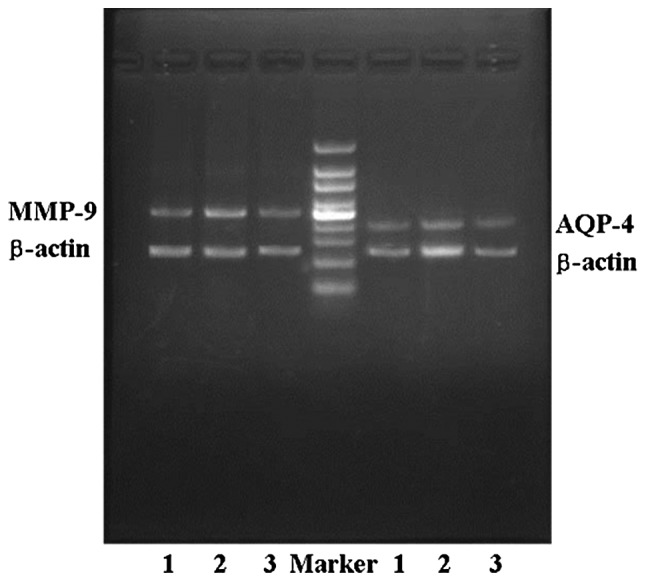
Expression of matrix metalloproteinase (MMP)-9 and aquaporin (AQP)-4 was detected using reverse transcription-polymerase chain reaction (RT-PCR). Lane 1, sham surgery group; lane 2, hypoxic ischemia (HI) group; lane 3, progesterone group.

Immunohistochemistry revealed that MMP-9-positive staining was manifested by buffy granules extensively distributed in the cerebral cortex and primarily located in the cytoplasm of neurons. In the sham surgery group, MMP-9 protein expression was low, with light-staining and sparsely distributed buffy-stained granules. The HI group demonstrated the highest level of MMP-9 protein expression, with deeply stained, densely distributed immunopositive granules. Although the MMP-9 expression level in the PROG group was high, it was noticeably lower than that in the HI group (P<0.05; Fig. 1A–C and Table I). The RT-PCR results 24 h after HI demonstrated that the MMP-9 mRNA expression level in the HI group was markedly higher than those in the sham surgery and PROG groups (P<0.05; Fig. 2 and Table I).

### Effect of PROG on AQP-4 expression in the cerebral cortex

Immunohistochemistry revealed that AQP-4-positive cells were round or oval, with stained membranes and cytoplasm. The HI group demonstrated significantly higher AQP-4 expression levels compared with the sham surgery group (P<0.01) and the PROG group (P<0.05; Fig. 1D–F and Table I). The RT-PCR results 24 h after HI demonstrated that the HI group had significantly higher AQP-4 mRNA expression levels than the sham surgery and the PROG groups (P<0.05; Fig. 2 and Table I).

### Effects of PROG on MMP-9 content, BBB permeability and cerebral edema in HIBD cerebral tissues

The MMP-9 content, BBB permeability and cerebral edema in the HI group were significantly higher compared with those in the sham surgery group (P<0.01) and the PROG group (P<0.05; Table II).

## Discussion

The preventive effect of PROG on brain ischemia has attracted extensive attention in recent years. It is synthesized and secreted in the nervous system and increases the synthesis of glucose transporters in cerebral tissues subjected to HI and accelerates the transmission of glucose towards neurons through the BBB. These functions improve the recovery of cerebral energy metabolism, reduce nerve cell apoptosis and reduce cerebral edema, thereby preventing HIBD (17–19). However, the associated underlying molecular mechanism and channel remain unclear. In the current study, we determined whether PROG alleviates BBB damage and cerebral edema by controlling the expression of AQP-4 and MMP-9.

BBB damage is one of the important pathophysiological mechanisms of ischemic brain damage. Cerebral edema caused by brain damage is a mixed type of edema closely correlated with increased BBB permeability (20). In the current study, the results demonstrated that the intraceptalic amount of EB dye and cerebral moisture capacity in the HI group were noticeably higher compared with those in the sham surgery group. This finding indicates that HI destroyed the BBB structure, thereby increasing its permeability and causing cerebral edema. The amount of EB and the cerebral moisture capacity in the PROG prophylaxis group were significantly lower compared with those in the HI group, which indicates that PROG has a cerebral protective effect. However, whether the molecular mechanism and channel underlying this effect depend on inhibition of the expression of AQP-4 and MMP-9 remains uncertain.

AQPs, a group of cell membrane transporters associated with moisture permeability, play critical roles in mediating transmembrane moisture flow. Among the AQPs, AQP-4 is the primary mediator of water flow in cerebral tissues. This AQP constitute the second BBB to regulate moisture transport (21). Changes in the intracephalic microenvironment following brain damage upregulate AQP-4 expression, which changes the cell membrane structures to increase BBB permeability and moisture permeability (22). In the current study, the HI group exhibited upregulated AQP-4 expression in the cerebral cortex and noticeable cerebral edema. These results suggest that HIBD upregulates AQP-4 expression to increase BBB permeability, leading to cerebral edema. Inhibiting AQP-4 expression decreases cerebral edema, thereby reducing the mortality and disability rates caused by nervous system diseases (23). AQP-4 knockout markedly alleviates the brain swelling caused by acute water intoxication or ischemic hemispheric stroke (24), which suggests that AQP-4 plays a critical role in the development of cerebral edema. This finding also indicates that inhibiting AQP-4 expression reduces or relieves the cerebral edema caused by AQP-4. Therefore, strategies to inhibit AQP-4 expression have become the core issue of post-brain damage edema improvement. In the current study, BBB permeability and cerebral moisture capacity were significantly reduced in the HIBD neonatal rats following ectogenic PROG administration. Furthermore, ectogenic PROG noticeably inhibited the mRNA and protein expression of AQP-4. These results suggest that PROG downregulates AQP-4 expression in the cerebral cortex of HIBD neonatal rats to reduce BBB damage and cerebral edema, which may be one of the mechanisms underlying the preventive effect of PROG on HIBD.

MMPs are metalloprotein incision enzymes that require calcium and zinc ions and they are responsible for extracellular matrix degradation. MMPs act on the components of vascular basement membranes to degrade the majority of the extracellular matrices, including type IV gelatin collagens, laminins and fibronectins (25). MMPs are closely correlated with blood-cerebrospinal fluid barrier dysfunction, which causes the outflow of a large amount of moisture, electrolytes and proteins. Such outflows result in the formation of vasogenic cerebral edema and damage to neurons, thereby aggravating ischemic brain damage. MMP-9 is the most important MMP in the nervous system. MMP-9, also called gelatinase B, is primarily synthesized and secreted by neutrophils, monocytes, macrophages, microglia, astrocytes and intravascular cells. This metalloprotein is involved in the degradation of almost all extracellular matrix components and destroys the structural integrity of vascular walls and the BBB (26). The present study also demonstrated increased MMP-9 expression and evident cerebral edema following HIBD. This finding indicates that MMP-9 destroys the BBB and promotes cerebral edema by degrading extracellular matrices. MMP-9 is only expressed at low levels in normal brain tissues. However, pathological conditions, including ischemia and inflammation, increase its transcription, subsequently increasing its protein expression. One of the causes of HIBD is a local inflammatory reaction. Inflammatory reactions activate MMP-9 to first degrade extracellular matrices and then eventually damage the BBB. Applying synthesized MMP-9 inhibitors prevents BBB opening and alleviates the severity of cerebral edema within 24 h, which suggests that BBB opening and cerebral edema formation are correlated with MMP-9 (27). The present study demonstrated that ectogenic PROG noticeably inhibits MMP-9 mRNA expression, and reduces the number of MMP-9-positive cells and the MMP-9 content in the cerebral cortex, thereby reducing BBB damage and cerebral edema.

In summary, administering ectogenic PROG attenuates BBB damage and cerebral edema in HIBD neonatal rats by downregulating the expression of AQP-4 and MMP-9 in the brain cortex. This effect may be one of the underlying mechanisms by which PROG prevents HIBD. Further studies are required to determine the mechanisms underlying the neuroprotective effect of PROG. Once these mechanisms are understood, PROG is likely to be used extensively as an effective neuroprotectant for neonatal HIBD in clinical practice.

## Figures and Tables

**Table I.** Effect of PROG on the expression of MMP-9 and AQP-4 in the brain tissue of neonatal rats.

Group	Immunohistochemistry		RT-PCR	
	
	MMP-9	AQP-4	MMP-9	AQP-4
Sham	0.241±0.021	0.263±0.034	0.37±0.03	0.36±0.02
HI	0.749±0.034^a^	0.658±0.028^a^	0.69±0.15^a^	0.74±0.14^a^
PROG	0.433±0.032^b^	0.410±0.032^b^	0.38±0.06^b^	0.41±0.12^b^

a P<0.05 vs. sham group;

b P<0.05 vs. HI and sham groups. PROG, progesterone; MMP, matrix metalloproteinase; AQP, aquaporin; RT-PCR, reverse transcription-polymerase chain reaction; HI, hypoxic ischemia.

**Table II.** Effects of PROG on MMP-9, BBB permeability and water content in the cerebral cortex following hypoxic-ischemic brain damage in neonatal rats.

Group	MMP-9 (ng/ml)	EB (*μ*g/g)	WC (%)
Sham	53.25±4.66	15.38±2.74	87.65±7.02
HI	89.43±9.48^a^	124.56±13.85^a^	93.23±10.52^a^
PROG	63.21±7.02^b^	95.41±8.57^b^	89.28±9.36^b^

Data are presented as mean ± standard deviation (n=8).

a P<0.05 vs. sham group;

b P<0.05 vs. HI and sham groups. PROG, progesterone; MMP, matrix metalloproteinase; BBB, blood-brain barrier; HI, hypoxic ischemia; EB, Evans blue; WC, water content.
